# A comparison of the time course of action and laryngeal mask airway insertion conditions with different doses of mivacurium for day-case urologic surgery in children: a prospective cohort study

**DOI:** 10.3389/fped.2024.1330737

**Published:** 2024-02-26

**Authors:** Hong Ye, Chunmiao Nian, Lijun Zhou, Yuman Xie, Fan Li, Tao Xue, Xueping Han

**Affiliations:** Department of Anesthesiology, Pain and Perioperative Medicine, The First Affiliated Hospital of Zhengzhou University, Zhengzhou, Henan, China

**Keywords:** mivacurium, day-case surgery, urologic surgery in children, laryngeal mask airway, anesthesiology

## Abstract

**Objective:**

To investigate the time course of action of different doses of mivacurium and determine the appropriate dose for laryngeal mask airway (LMA) insertion for day-case urologic surgery in children.

**Methods:**

A total of 105 patients who enrolled in this study between March 2021 and December 2021 were randomised into 3 groups: Group A (mivacurium 0.15 mg/kg, *n* = 35), Group B (mivacurium 0.20 mg/kg, *n* = 35) and Group C (mivacurium 0.25 mg/kg, *n *= 35). The different doses of mivacurium were injected before LMA insertion. The primary outcomes included the grading of conditions for the LMA insertion-18 score, onset time, recovery index and the duration that mivacurium was effective. Secondary outcomes included pulse oxygen saturation, mean blood pressure, heart rate and the incidence of adverse events.

**Results:**

The score of the conditions for LMA insertion in Group A was significantly lower than in Groups C and B (*p* < 0.005). There was a significant difference in the onset time between Groups B and A (*p* < 0.005). There was no significant difference in the overall incidence of adverse reactions between these groups (*p* > 0.05).

**Conclusion:**

Anaesthesia with 0.2 mg/kg of mivacurium can effectively shorten the onset time and facilitate insertion of the LMA in children undergoing day-case urologic surgery.

## Introduction

Enhanced recovery after surgery (ERAS) has gradually gained popularity, especially in day-case surgery for paediatric patients (1). Therefore, in day-case surgery, non-depolarising neuromuscular blocking agents (NMBs) with a rapid onset and short duration are required for brief surgical procedures and endotracheal intubation or laryngeal mask insertion. Mivacurium, a bisbenzyltetrahydroisoquinolinium compound, is a short-acting, non-depolarising neuromuscular blocker that is quickly hydrolysed by plasma cholinesterase (2). Paralysis can rapidly recover after mivacurium, which means the use of reversal agents after the administration of mivacurium in normal patients is usually not necessary (3). This enhancement of a neuromuscular block has obvious advantages for day-case surgery anaesthesia (4).

When using mivacurium for intubation in newborns, effective bag and mask ventilation can easily be achieved due to muscle relaxation and is associated with excellent intubation conditions, resulting in a high success rate (5, 6). Mivacurium has many merits, such as not impacting the bispectral index (BIS) and cerebral state index values in paediatric patients anaesthetised with propofol (7), insignificant cumulation (8) and an almost constant rate of recovery, irrespective of the total dose administered and mode of administration, such as bolus or infusion (9). However, it exhibits dose-dependent adverse effects on the activation of mast cells, which release histamine. A single large dose and rapid injection can result in capillary dilation, skin flushing and even laryngeal spasms (10). Nevertheless, the effect of different dosages of intravenous mivacurium administration in day-case urologic surgery for children of different ages using a laryngeal mask airway (LMA) has rarely been reported.

An LMA is a device that is inserted into the mouth and throat to provide a seal around the laryngeal inlet and allow ventilation without the need for endotracheal intubation (11). It has several advantages over endotracheal intubation, such as less airway trauma, a lower incidence of postoperative sore throat, cough and laryngospasm and shorter insertion and removal times (12). However, LMA insertion may also cause adverse airway reflexes, such as coughing, gagging and airway obstruction, which can compromise the quality and safety of anaesthesia and surgery (13). Therefore, some degree of muscle relaxation may be beneficial to facilitate LMA insertion and reduce the haemodynamic response and patient discomfort (14). Moreover, some types of urologic surgery, such as laparoscopy, may require a deeper level of anaesthesia and muscle relaxation to ensure adequate surgical conditions and prevent intraoperative movement. Therefore, we believe that the use of NMBs with LMA is beneficial in some cases of paediatric urologic surgery, especially when the surgery is expected to last longer than 15 min or involves laparoscopy.

The use of muscle relaxants for LMA insertion is not contraindicated, but rather, a matter of debate and preference among anaesthesiologists. Some studies have shown that muscle relaxants can facilitate LMA insertion and reduce the incidence of adverse airway reflexes, such as coughing, gagging and laryngospasm (15, 16). However, other studies have suggested that muscle relaxants are not necessary for LMA insertion and may increase the risk of residual neuromuscular block and postoperative complications (17, 18). Therefore, the choice of muscle relaxant and its dose should be individualised according to the patient's characteristics, the type and duration of surgery and the availability and cost of the drug.

The present study investigated the time course of action of different doses of mivacurium and determined the appropriate dose of mivacurium for LMA insertion for day-case urologic surgery in children.

## Materials and methods

### Research objectives

This prospective, single-blinded and randomised controlled clinical trial was approved by the Ethics Committee (2020-KY-0314-002) of the First Affiliated Hospital of Zhengzhou University (Henan, China) and complied with the 1964 Helsinki Declaration and its later amendments. It was registered with the Chinese Clinical Trials Registry (ChiCTR2300068311). In this study, 150 children of both genders were screened at the hospital between March 2021 and December 2021. The inclusion criteria were as follows: aged 3–10 years; an American Society of Anaesthesiologists (ASA) physical status of I–II; no abnormalities in laboratory and imaging examinations before the operation; were scheduled for urologic surgery under general anaesthesia. Subsequently, after written informed consent from parents or guardians had been obtained, 105 children were enrolled in the study.

The exclusion criteria were as follows: patients with a respiratory infection, nervous system or cardiovascular diseases or impaired kidney or liver function; patients with hypersensitivity to mivacurium or other anaesthetic agents; patients with neuromuscular or metabolic diseases; patients who had been administered muscle relaxant during the preceding 7 days or participated in any clinical tests within the previous 30 days; patients receiving antibiotics that affected NMBs; patients who received laryngeal mask placement times ≥3 times and/or poor placement of the LMA; patients with a 20% deviation from the ideal child body weight [calculated by the formula: weight in kg = 3 × (age in years + 5)].

### Research grouping

An investigator who was neither involved in the clinical care nor the follow-up procedures enrolled participants and subsequently generated the random allocation sequence. A computer-generated random number randomised the children into 3 groups. Neuromuscular block was achieved by ×1.5 (0.15 mg/kg) times the effective mivacurium dose in 95% of the population (ED_95)_ for Group A, ×2.0 (0.20 mg/kg) ED_95_ of mivacurium for Group B and ×2.5 (0.25 mg/kg) ED_95_ of mivacurium for Group C. Random allocation was achieved using sealed opaque envelopes, which were opened by the investigator immediately before the induction of anaesthesia. Anaesthesiologists were not blind to the group assignment, as they needed to administer the medication accordingly, but the follow-up investigators and the patients were blind to the type of induction they would receive when they gave consent to be included in the study.

### Anaesthesia and laryngeal mask airway insertion

All children received 0.5 mg/kg esketamine premedication or nasal spray. When the weight of children was >20 kg, 10 mg of esketamine nasal spray was given. Routine monitoring was conducted upon arrival in the operating room, including an electrocardiogram, pulse oxygen saturation (SpO_2_), non-invasive blood pressure and the BIS. The temperature was measured over the adductor pollicis muscle and maintained between 36°C–37.5°C. The induction of general anaesthesia was performed as follows: after establishing intravenous access, intravenous fentanyl 3.0–5.0 μg/kg was administered. Then, 2 min later, propofol 2 mg/kg was injected. Next, neuromuscular block in the children was monitored using acceleromyography employing a TOF-Watch SX machine (Organon, Ireland), with supramaximal stimulation of the ulnar nerve being achieved at the wrist by 2 surface electrodes placed 2 cm apart. The acceleration indicator was fixed to the volar aspect of the distal part of the thumb. The ulnar nerve was stimulated at the wrist by train-of-four (TOF) stimulation (2 Hz, 0.2 ms) every 12 s and neuromuscular function was measured at the adductor pollicis. Mivacurium was given according to the group assignment, at an infusion speed within 30–60 s to achieve a rapid onset of neuromuscular block and to minimise the risk of histamine release and associated cardiovascular effects, which may occur with faster injection speeds. According to the age of the children, different-sized LMAs were inserted by an experienced anaesthesiologist when maximum depression occurred. Anaesthesia was maintained with propofol (4–10 mg/kg/h) and remifentanil (0.3–0.5 μg/kg/min). Ventilation was adjusted to maintain partial pressure of end-tidal CO_2_ between 30 and 40 mmHg. All anaesthetic agents were stopped when the operation procedure was completed and the anaesthesiologist judged the time of withdrawal of the LMA. After the LMA was removed, the children were transferred to the post-anaesthesia care unit (PACU) for recovery. None of the patients needed to be reversed with neostigmine at the end of the procedure, as they all achieved complete recovery from the neuromuscular block as assessed by the TOF ratio [(TOFr) > 0.9].

### Observation indexes

The primary outcome measurements of this clinical experiment were the grading of conditions for LMA insertion-18 using 6 variables as follows: ease of LMA insertion, jaw opening, coughing, gagging, airway obstruction and patient movements; each variable was graded on a 3-point scale (19). The following neuromuscular effects were measured: the onset time (time between the injection of mivacurium and more than 95% depression of the first twitch of the TOFr); the duration of clinical action (time from the beginning of the injection of mivacurium to 25% recovery of the TOFr); and the recovery index (RI) (time of the TOFr from 25% to 75% recovery of the first twitch).

The secondary efficacy indicators included SpO_2_, heart rate (HR), mean blood pressure (MBP) at different times, incidences of adverse events in the PACU and postoperative pain scores. The detection time points of SpO_2_, HR and MBP were 2 min before anaesthesia induction (T1), 30 s after loss of responsiveness (T2), 30 s after LMA insertion (T3), the end of surgery (T4) and 30 s after removing the LMA (T5). Among these, data recorded at 2 min before anaesthesia induction were regarded as the baseline. Adverse events that occurred during anaesthesia recovery in the PACU, which included hypoxaemia, skin flushing, nausea, vomiting and postoperative emergence agitation, were recorded. Hypoxemia was defined as SpO_2_ < 95%.

### Statistical analysis

The sample size was calculated according to the RI. Based on data from previous research (20), the RIs in children were 4 ± 2 min, *α* = 0.05 and *β* = 0.1. The sample size was calculated using PASS 15 software (NCSS, Kaysville, UT, USA). According to a 1:1:1 parallel control study, 27 patients in each group were required for this clinical trial; however, the sample size in each group was expanded to 35 patients to allow for those who failed to follow up. All the data in this study were analysed using the SPSS 26.0 software (IBM Corp., Armonk, NY, USA), and the measurement data were expressed by mean ± standard deviation (*χ* ± *s*). Quantitative variables were tested for normality with the Shapiro–Wilk test. The comparison among groups was performed using a one-way analysis of variance. The Mann–Whitney *U*-test was used for continuous variables with repeated measures. Qualitative data were presented as number (*n*) and percentage (%) and analysed using the chi-square test. Significance levels of *α* = 0.05 and *p* < 0.05 indicated that the difference was statistically significant.

## Results

### Patients

**Figure 1:**
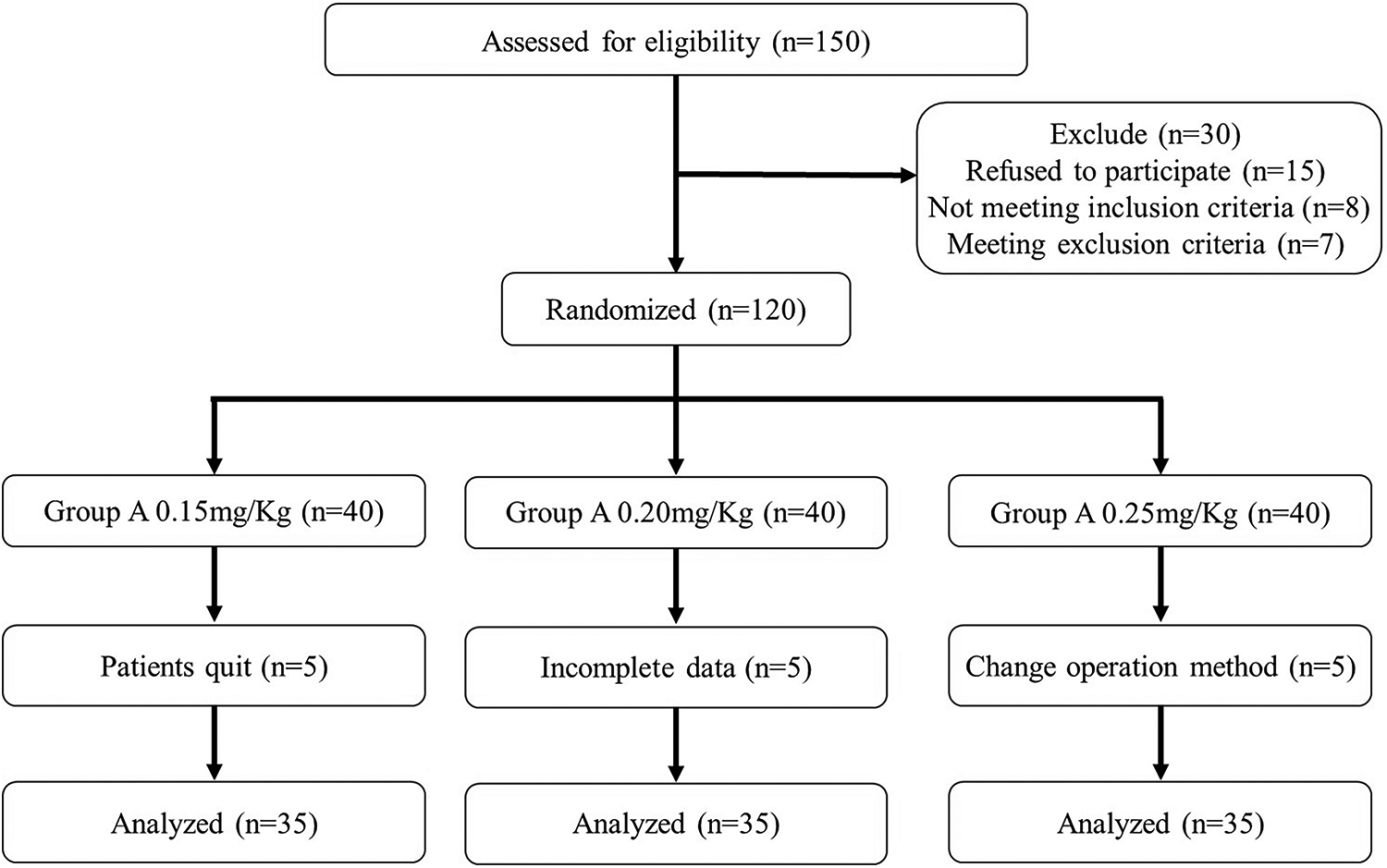
Flow diagram.

From March 2021 to December 2021, 150 children were initially screened for eligibility, 30 of whom were excluded and 120 were randomly allocated into 3 groups. Subsequently, 5 children's data were incomplete, 5 parents refused to participate in the study and the surgery modes of another 5 children were changed. The final analysis was performed based on data of 105 children. More details are shown in the flow diagram (Figure 1). The demographic and clinical characteristics of the 3 groups were comparable and are detailed in Table 1.

**Table 1:** Patient demographic and clinical parameters (*n* = 35 in each group).

Characteristics	Group A	Group B	Group C	*P* value
Age (years)	6 ± 4	6 ± 3	7 ± 3	0.163
Sex (male/female)	30/5	24/11	28/7	0.210
BMI (kg/m2)	17.3 ± 0.4	17.1 ± 0.4	18.0 ± 0.4	0.304
ASA grade (I/II)	32/3	33/2	32/3	0.873
Surgical time (min)	23.3 ± 4.6	23.5 ± 3.1	25.0 ± 3.7	0.124
Operation category				0.536
Ureteroscopic ureteral stent removal	12 (34.3%)	15 (42.9%)	13 (37.1%)	
Laparoscopic upper ligation of sheath process	8 (22.9%)	10 (28.6%)	10 (28.6%)	
Urethroplasty	10 (28.6%)	7 (20%)	8 (22.9%)	
Repair of urethral fistula	5 (14.3%)	3 (8.6%)	4(11.4%)	

Quantitative variables were presented as mean ± standard deviation (*χ* ± *s*) and tested for normality with Shapiro–Wilk test. The comparison among groups was performed using one-way analysis of variance (one-way ANOVA). Qualitative data were presented as number (*n*) and percentage (%) and analyzed by Chi-Square test. The significance level of *p* < 0.05 indicated that the difference was statistically significant. No statistically significant differences between groups were noted.

BMI, body mass index; ASA, American Society of Anesthesiologists.

There were 97 children of ASA status Ⅰ and 8 children of status 2161;. The children were aged 3–10 years, with an average age of (6.18 ± 2.21) years and a body mass index (BMI) of 12.7–23.7, with an average BMI of 17.47 ± 2.37. Regarding the types of surgery, these included 40 cases of ureteral stent removal, 28 cases of laparoscopic upper ligation of sheath process, 25 cases of urethroplasty and 12 cases of urethral fistula repair.

### Primary outcomes

Table 2 shows that all the children had an LMA inserted successfully at either the first or second attempt; among them, 2 (5.7%) children in Group A had 2 attempts. Three (8.6%) children in Group A and 1 (2.9%) child in Group B experienced partial jaw opening and 1 (2.9%) child in Group A experienced a slight cough. In terms of the patients moving, 4 (11.4%) children in Group A and 1 (2.9%) child in Group B experienced moderate movements. Even though there were no statistically significant differences in the 6 variables, the total score of Group A (17.77 ± 0.55) was significantly lower compared with Groups C (18.00 ± 0.00) and B (17.94 ± 0.24) (*p = *0.018), indicating that 0.15 mg/kg of mivacurium provided worse conditions for LMA insertion than 0.2 or 0.25 mg/kg of mivacurium. This may be due to insufficient muscle relaxation, partial jaw opening, coughing or patient movement in some cases of Group A. The total scores of Groups B and C were not significantly different (*p* = 0.153), indicating that 0.2 and 0.25 mg/kg of mivacurium provided similar and optimal conditions for LMA insertion.

**Table 2:** Ease of LMA insertion, Jaw opening, coughing, gagging, patient movements, airway obstruction and total score during anesthesia (*n* = 35 in each group).

** **	Grade	Description	Group A	Group B	Group C	*P* value
Ease of LMA insertion	3	Easy	33 (94.3%)	35 (100%)	35 (100%)	0.133
	2	Difficult	2 (5.7%)	0	0	
	1	Impossible	0	0	0	
Jaw opening	3	Full	32 (91.4%)	34 (97.1%)	35 (100%)	0.166
	2	Partial	3 (8.6%)	1 (2.9%)	0	
	1	Nil	0	0	0	
Coughing	3	Nil	34 (97.1%)	35 (100%)	35 (100%)	0.371
	2	Slight	1 (2.9%)	0	0	
	1	Severe	0	0	0	
Gagging	3	Nil	35 (100%)	35 (100%)	35 (100%)	–
	2	Slight	0	0	0	
	1	Severe	0	0	0	
Patient movements	3	Nil	31 (88.6%)	34 (97.1%)	35 (100%)	0.066
	2	Moderate	4 (11.4%)	1 (2.9%)	0	
	1	Vigorous	0	0	0	
Airway obstruction	3	Nil	35 (100%)	35 (100%)	35 (100%)	–
	2	Partial	0	0	0	
	1	Tatol	0	0	0	
Total score			17.77 ± 0.55	17.94 ± 0.24	18.00 ± 0.00	0.018

Quantitative variables were presented as mean ± standard deviation (*χ* ± *s*) and tested for normality with Shapiro–Wilk test. The comparison among groups was performed using one-way analysis of variance (one-way ANOVA). Qualitative data were presented as number (*n*) and percentage (%) and analyzed by Chi-Square test. The significance level of *p *< 0.05 indicated that the difference was statistically significant. LMA, laryngeal mask airway.

The onset time, time of clinical action and RI were compared among the three groups and are shown in Table 3. There were no significant differences in the onset time between Groups B and C (95% confidence interval [CI] = (−0.161, 0.789), *p* = 0.295) and both groups had significantly shorter onset times than Group A (95% CI = [−1.263, −0.51], *p *= 0.029% and 95% CI = [−1.543, −0.400], *p *= 0.000, respectively). There were no significant differences in the time of clinical action among the three groups (95% CI = [−1.926, 0.371], *p *= 0.308; 95% CI = [−2.163, 0.134], *p *= 0.102; 95% CI = [0.643, 2.940], *p* = 0.001). There were also no significant differences in RI between Groups A and B [95% CI = (−1.233, 0.473), *p *= 0.843] and between Groups B and C [95% CI = (−1.433, 0.273), *p *= 0.303]. However, Group C had a significantly larger RI than Group A [95% CI = (0.107, 1.813), *p *= 0.022].

**Table 3:** Onset time, duration of clinical action, recovery index, extubation time and the length of PACU (min) in different group (*n* = 35 in each group).

** **	Group A	Group B	Group C	*F*	*P* value
Onset time (min)	3.46 ± 1.16	2.80 ± 0.88	2.49 ± 0.73	9.65	0.000
Duration of clinical action (min)	10.99 ± 2.01	11.77 ± 2.18	12.78 ± 1.69	7.25	0.001
Recovery index (min)	4.21 ± 1.58	4.59 ± 1.47	5.17 ± 1.34	3.81	0.025

Quantitative variables were presented as mean ± standard deviation (*χ* ± *s*) and tested for normality with Shapiro–Wilk test. The comparison among groups was performed using one-way analysis of variance (one-way ANOVA). The significance level of *p *< 0.05 indicated that the difference was statistically significant. PACU, post anaesthesia care unit.

### Secondary outcomes

**Figure 2:**
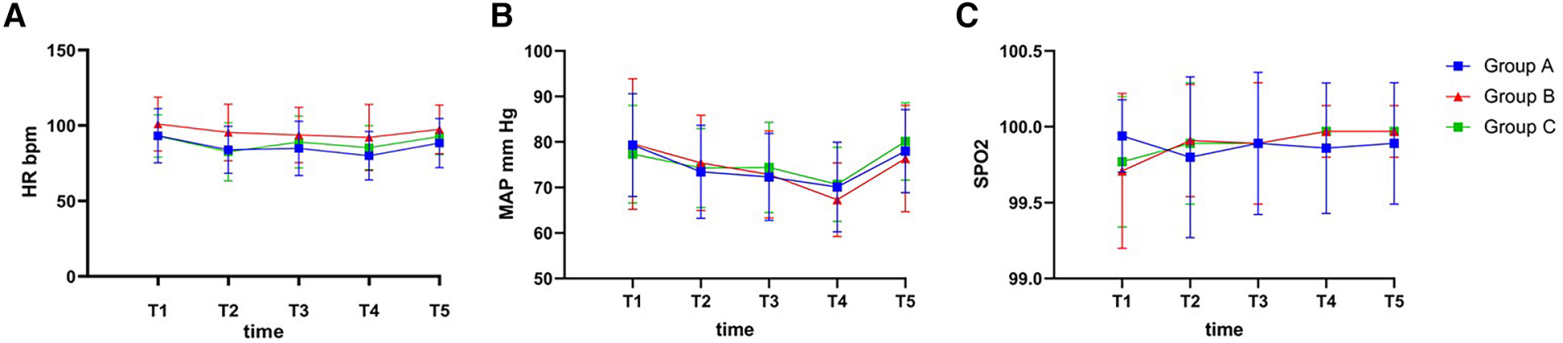
The intraoperative hemodynamic changes. (**A**) MAP during the anesthesia; (**B**) HR during the anesthesia; (**C**) SPO2 during the anesthesia. MAP, mean arterial pressure; HR, heart rate; SPO2, saturation of O2 percutaneously; T1, 2 min before anesthesia induction; T2, 30 s after loss of responsiveness; T3, 30 s after LMA insertion; T4, the end of surgery; T5, 30 s after removing LMA. *p* < 0.05 was defined as statistically significant.

Figure 2 shows the HR, average blood pressure and SpO_2_ trend at 5 different times. At time T2, the HRs of Groups A (83.91 ± 15.59) and C (82.60 ± 19.25) were reduced compared with Group B (95.40 ± 18.72) (95% CI = [−21.92, −1.06], *p* = 0.026% and 95% CI = [−23.23, −2.37], *p* = 0.011, respectively). Compared with Group B (92.14 ± 22.10 and 97.46 ± 16.10), Group A (79.94 ± 15.99 and 88.31 ± 16.27) had a lower HR during the injection study at the T4 and T5 time points (95% CI = [−22.59, −1.81], *p* = 0.016% and 95% CI = [−17.81, −0.47], *p* = 0.035, respectively). Furthermore, the MBP and SpO_2_ values of each group were similar, with no significant differences among the 3 groups at each time point during the anaesthesia (*p *> 0.05), despite significant differences being observed in the intragroup comparisons before and after induction (*p* < 0.05).

Table 4 shows that adverse reactions occurred in 5 cases/times (14.3%) in Group A, 4 cases/times (11.4%) in Group B and 9 cases/times (25.7%) in Group C. There was no significant difference in the overall incidence of adverse reactions between the 3 groups (*p* > 0.05). The incidence of hypoxaemia in the PACU included 1 patient (2.9%), 0 patients (0%) and 1 patient (2.9%) in the 3 groups, respectively, but no significant difference was observed between the 3 groups (*p* > 0.05), which were corrected by oxygen inhalation through a facial mask. Notably, 1 case (2.9%) in Group C experienced vomiting during the PACU stay, which was controlled by adding 0.35 mg/kg dolasetron; this was not experienced by any other patients in the other groups. None of the children in the 3 groups experienced nausea. In this study, skin flushing and somnolence were adverse reactions with a slightly higher incidence; 1 case (2.9%) in Group A, 2 cases (5.7%) in Group B and 4 cases (11.4%) in Group C experienced skin flushing during the operation, and 3 cases (8.6%) in Group A, 2 cases (5.7%) in Group B and 3 cases (8.6%) in Group C experienced somnolence during the PACU stay, which may have been related to the transient histamine release induced by mivacurium and drug residue from general anaesthesia. Through close observation and monitoring, all of the Steward scores of children reached above 5 points and they were sent back to the ward. None of the children displayed bronchospasm, which is a rare but serious adverse reaction of mivacurium-induced histamine release.

**Table 4:** Adverse events in anesthesia recovery period (*n* = 35 in each group).

Adverse events	Group A	Group B	Group C	*P* value
Hypoxemia	1 (2.9%)	0 (0.0%)	1 (2.9%)	0.601
Skin flushing	1 (2.9%)	2 (5.7%)	4 (11.4%)	0.343
Nausea	0 (0.0%)	0 (0.0%)	0 (0.0%)	–
Vomiting	0 (0.0%)	0 (0.0%)	1 (2.9%)	0.364
Somnolence	3 (8.6%)	2 (5.7%)	3 (8.6%)	0.873
Total	5 (14.3%)	4 (11.4%)	9(25.7%)	0.245

Qualitative data were presented as number (*n*) and percentage (%) and analyzed by Chi-Square test. The significance level of *p < 0.05 indicated that the difference was statistically significant. No statistically significant differ.

## Discussion

Day-case surgery has been widely adopted by many medical staff and patients as it is more cost-effective than traditional inpatient surgeries. It has become the focus of medical staff to take effective measures for rapid anaesthesia awakening. This study showed that the grading of conditions for the LMA insertion-18 score of 0.15 mg/kg mivacurium was lower than 0.2 mg/kg and 0.25 mg/kg mivacurium in children during ambulatory urologic surgery. In addition, the onset times of 0.2 and 0.25 mg/kg mivacurium were shorter than 0.15 mg/kg mivacurium, which was consistent with the results of Zeng et al. (2). However, the duration of clinical action and RI of a medium induction dose of mivacurium did not prolong or shorten the onset times compared with a small or increasing dose. Moreover, the overall incidence of adverse reactions in 0.2 mg/kg doses of mivacurium was higher than in 0.25 mg/kg doses, although there were no statistically significant differences. Therefore, mivacurium has not only a faster effect, shorter duration of action and no accumulation but also a faster recovery time, which means it could be more conducive to obtaining good intubation conditions and providing good surgical conditions in urologic surgery for children, especially 0.2 mg/kg mivacurium.

In recent years, mivacurium, which has a short elimination half-life, has been widely used in clinical general anaesthesia procedures in day-case surgery in China (2, 10, 21). Because it has a shortened waiting time after deep neuromuscular blockage compared with intermediate-acting cisatracurium and rocuronium, no direct effect on liver and kidney functions, rapid postoperative recovery, no accumulative effect in the body and a slight effect on circulation (9, 10), mivacurium has become the main muscle relaxant used for paediatric anaesthesia in some countries (4). As such, mivacurium's pharmacodynamic and pharmacokinetic characteristics are consistent with day-case urologic surgery in children. Recent clinical studies on day-case urologic surgery in children were mostly focused on the selection of NMBs, with less attention paid to the appropriate dose requirements for mivacurium in anaesthesia induction.

This prospective, double-blinded, randomised controlled clinical trial compared 3 different doses of mivacurium in anaesthesia induction during ambulatory urologic surgery in children. The results suggest that anaesthesia induction with 0.2 mg/kg of mivacurium not only entails a shorter onset time than 0.15 mg/kg but also foreshortens the extubation time and the length of PACU stay compared with 0.25 mg/kg of mivacurium. Therefore, the authors deduced that anaesthesia induction with 0.20 mg/kg of mivacurium is more suitable for day-case urologic surgery than 0.15 mg/kg and 0.25 mg/kg. The focus of this study was to better understand the clinical use of mivacurium by exploring the suitable patient and operation category, the indication and reasonable compatibility of mivacurium and discovering its optimum usage method and dosage to fully exert the muscle relaxation effect and avoid postoperative residual neuromuscular blockage and adverse reactions. The results demonstrated that increasing the dose of mivacurium significantly improved the grading of conditions for LMA insertion.

In this study, 7 children (1 case in 0.15 mg/kg, 2 cases in 0.2 mg/kg and 4 cases in 0.25 mg/kg) were observed to experience slight skin flushing without a significant change in haemodynamics after anaesthesia induction. A moderately slow injection speed of 30–60 s was used, which was different from that used in the research conducted by Burburan (22). Fortunately, the slight skin flushing of these children gradually subsided after close observation and monitoring. It is possible that pretreatment with H1/H2 antagonists, or with promethazine, could reduce the effects of mivacurium-induced histamine release and provide stable haemodynamics during the administration of anaesthesia (23, 24). In addition, esketamine nasal spray was used to reduce preoperative separation anxiety, which was also reported to reduce the incidence of postoperative emergence agitation in children (25); however, whether there is a correlation between the use of esketamine and the somnolence experienced by 8 children in this study remains unknown. Persistent neuromuscular block is a potential risk of mivacurium, particularly in patients with atypical plasma cholinesterase or the concomitant use of other drugs that may prolong the action duration of mivacurium (26). Persistent neuromuscular block can lead to postoperative respiratory complications, such as hypoxemia, atelectasis and pneumonia (27). Therefore, it is essential to monitor neuromuscular functioning and assess patients for complete recovery from the neuromuscular block before extubation and discharge. This study used the TOFr as the objective indicator of neuromuscular recovery, and the authors ensured that all patients had a TOFr of more than 0.9 before removing the LMA and transferring them to the PACU. This is consistent with the current recommendations for the management of neuromuscular block (21). It has been reported that the incidence of atypical cholinesterase is much lower in Chinese (0.02%) than in Western populations (0.5%), which may explain why mivacurium is more frequently used for outpatient surgery in China than in the West (28). However, this does not eliminate the possibility of encountering patients with atypical cholinesterase or other factors that may affect the elimination of mivacurium, such as renal or hepatic impairment, age or drug interactions. Therefore, it is still advisable to monitor the neuromuscular function and ensure the complete recovery from neuromuscular block in all patients receiving mivacurium. In addition to mivacurium, other drugs that can reduce adverse airway reflexes during laryngeal mask insertion include dexmedetomidine and lidocaine. Dexmedetomidine is a selective alpha-2 adrenergic agonist that has sedative, analgesic and sympatholytic effects and can attenuate the haemodynamic response to laryngeal mask insertion (27). Lidocaine is a local anaesthetic that can be applied topically or intravenously to suppress the cough reflex and reduce the cardiovascular response to laryngeal mask insertion (29). However, these drugs may also have drawbacks, such as bradycardia, hypotension, prolonged sedation or allergic reactions (30). Therefore, the choice of the optimal drug for laryngeal mask insertion should be based on the individual characteristics of the patient, the type and duration of the surgery and the availability and cost of the drug.

Several limitations should be noted in this study. First, the postoperative follow-up of children was not continued, and the influence on the recovery of the pulmonary function and the occurrence of complications was not observed. Second, the participants in the research were older children; younger children were not included due to their immature cardiovascular and respiratory systems. Therefore, further studies should be performed to investigate whether younger children are suitable for the recommended induction dose of mivacurium. Third, the authors also observed some movements or a need for a second attempt to insert the LMA in some cases, even with high doses of mivacurium and ToF monitoring. This may have been due to individual variations in the pharmacodynamics and pharmacokinetics of mivacurium, the optimal timing of LMA insertion or the experience of the anaesthesiologist. Further studies are needed to clarify these factors and optimise the conditions for LMA insertion with mivacurium. Finally, this study did not compare the authors' anaesthesia plan with other alternatives, such as the inhalation of sevoflurane alone or with minimal intravenous drugs, which may be simpler or more suitable for day-case urologic surgery in children. Future studies are needed to evaluate the optimal anaesthesia plan for this type of surgery.

In summary, 0.2 mg/kg of mivacurium in anaesthesia induction during day-case urologic surgery in children can reduce the onset time and foreshorten the extubation time and the length of PACU stay.

## Data Availability

The original contributions presented in the study are included in the article/Supplementary Material, further inquiries can be directed to the corresponding author.
